# Distribution of peri-device leaks on transoesophageal echocardiography and cardiac computed tomography after WATCHMAN implantation: a multicentre core-lab analysis

**DOI:** 10.1093/europace/euag128

**Published:** 2026-05-26

**Authors:** Issam Motairek, Mohamad Mdaihly, Walid Saliba, Andrea Natale, Rodney Horton, Devi Nair, Bradley J Mikaelian, Manish Shah, Ashish Sadhu, Kathy Wolsky, Jay Ramchand, Wael A Jaber

**Affiliations:** Heart, Vascular & Thoracic Institute, Cleveland Clinic, Cleveland, OH, USA; Heart, Vascular & Thoracic Institute, Cleveland Clinic, Cleveland, OH, USA; Heart, Vascular & Thoracic Institute, Cleveland Clinic, Cleveland, OH, USA; Cardiology Department, Texas Cardiac Arrhythmia Institute, Austin, TX, USA; Cardiology Department, Texas Cardiac Arrhythmia Institute, Austin, TX, USA; Cardiology Department, St. Bernards Medical Center & Arrhythmia Research Group, Jonesboro, AR, USA; Cardiology Department, UCHealth Heart Clinic – Memorial Hospital Central, Colorado Springs, CO, USA; Cardiology Department, MedStar Washington Hospital Center, Washington, DC, USA; Cardiology Department, Phoenix Cardiovascular Research Group, Phoenix, AZ, USA; Heart, Vascular & Thoracic Institute, Cleveland Clinic, Cleveland, OH, USA; Heart, Vascular & Thoracic Institute, Cleveland Clinic, Cleveland, OH, USA; Heart, Vascular & Thoracic Institute, Cleveland Clinic, Cleveland, OH, USA

**Keywords:** Peri-device leak, Left atrial appendage occlusion, Watchman, Transoesophageal echocardiography, Cardiac tomography

Left atrial appendage occlusion (LAAO) has emerged as an alternative stroke prevention strategy in patients with non-valvular atrial fibrillation and certain contraindications to oral anticoagulation.^1^ Imaging surveillance after LAAO has guided antithrombotic de-escalation decisions. In the landmark WATCHMAN trials, standard framework relied on transoesophageal echocardiography (TEE) at 6 weeks following device implantation to assess peri-device leak (PDL) and device-related thrombus (DRT) before discontinuing anticoagulation.^1^ However, this technique is operator-dependent and can cause patient discomfort. Recently, cardiac computed tomography (CT) has emerged as a non-invasive alternative, offering high-resolution imaging that detects residual patency more frequently.^2,3^ However, real-world data on the anatomical distribution of PDL across modalities remain poorly characterized, and standardized methods to describe leak distribution are lacking. We report a multicentre core-laboratory analysis of PDL size and anatomical localization using standardized clock-face mapping on both TEE and cardiac CT.

Patients undergoing WATCHMAN FLX or FLX Pro implantation were enrolled in a prospective multicentre registry and followed per local standard of care. Transoesophageal echocardiography was performed at implant and/or first follow-up (45–90 days), while cardiac CT was performed at first follow-up (45–90) days. All imaging studies were interpreted by a central imaging core laboratory. Study was approved by the institutional review board. Procedural guidance was performed under TEE in the majority of cases (80.7%), with intracardiac echocardiography (ICE) used in the remaining 19.2% of procedures (2D ICE: 17.2%; 4D ICE: 2.0%). In a subset of cases (22.8%), both TEE and ICE were used concurrently.

On TEE, PDLs were defined by colour Doppler flow and classified as <3 mm or ≥3 mm. Locations were recorded as posterior, anterior, inferior, mitral, limbus, or through-face. A clock-face model was used for visualization. On CT, PDLs were defined as contrast opacification within the LAA and categorized by minimum width (<3 mm or ≥3 mm). Leak locations were mapped using a 12-point clock-face centred on the LAA ostium, with 12:00 representing anterior and 6:00 posterior, following standard axial CT orientation.

Distribution of peri-device leak locations on TEE and cardiac CT is shown in ***Figure 1***. Among 974 patients undergoing post-implant TEE, 67 (6.9%) had PDLs, of whom 57 (85.1%) were <3 mm. In this subgroup, the majority were posterior 38 (66.7%), followed by limbus 8 (14.0%), inferior 5 (8.8%), anterior 5 (8.8%), and mitral 1 (1.8%). Among the remaining 10 (14.9%) patients with leaks ≥3 mm, most were posterior 5 (50.0%), followed by limbus 3 (30.0%) and inferior 1 (10.0%). At first follow-up, 228 (24.9%) patients had PDLs. Of these, 153 (69.2%) were <3 mm, most commonly posterior 68 (44.4%), followed by anterior 30 (19.6%), limbus 29 (19.0%), inferior 15 (9.8%), and through-face 9 (5.9%). Among 68 (30.8%) patients with leaks ≥3 mm, the posterior location remained most frequent 31 (45.6%), followed by anterior 14 (20.6%), limbus 10 (14.7%), inferior 9 (13.2%), and through-face 4 (5.9%).

**Figure 1:**
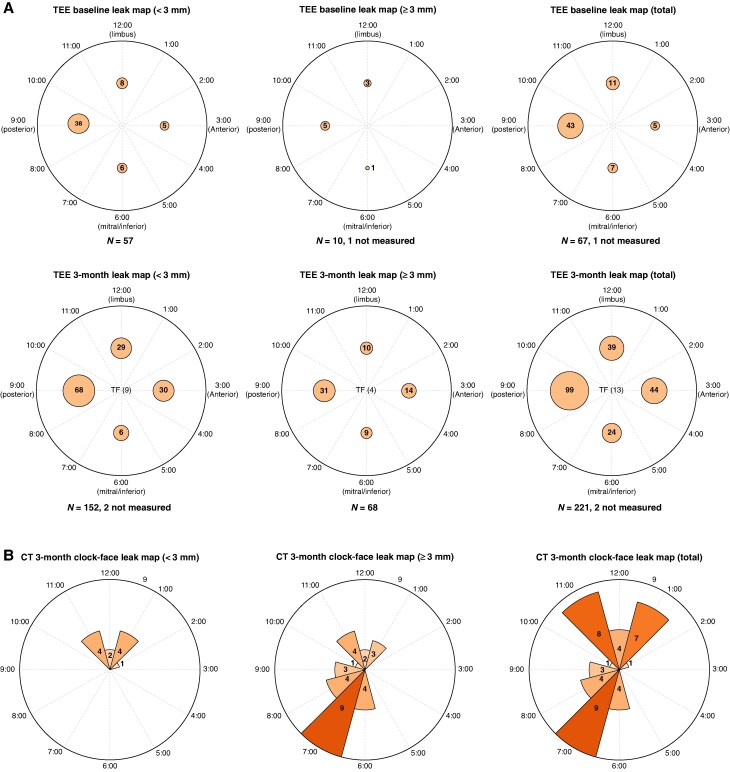
Distribution of peri-device leak (PDL) locations on transoesophageal echocardiography (TEE) and cardiac computed tomography (CT) following WATCHMAN implantation. (*A*) Clock-face bubble maps of TEE-detected PDLs at baseline (*N* = 67) and 3-month follow-up (*N* = 221), stratified by size (<3 mm, ≥3 mm, and all leaks). Circle size is proportional to leak count; clock-face orientation: 12 o'clock = limbus, 6 o'clock = mitral/inferior, 3 o'clock = anterior, 9 o'clock = posterior. (*B*) Clock-face rose diagrams of CT-detected PDLs at 45–90 days post-implantation (*N* = 41), stratified by size (<3 mm, ≥3 mm, and all leaks); wedge length is proportional to leak count. Leaks were predominantly posterior across both modalities and time points. PDL, peri-device leak; TEE, transoesophageal echocardiography; CT, computed tomography.

Six hundred and seventy-six patients were evaluated for the presence of PDLs using CT imaging. Forty-one (6.1%) had core-lab adjudicated PDLs with defined anatomical location and size. A total of 42 leaks were identified, including 1 patient with 2 distinct leak sites. Among 11 (26.8%) patients with leaks <3 mm, most leaks were located at 1:00 and 11:00 (4/11 each), followed by 2 (18.2%) at 12:00 (18.2%), and 1 (9.1%) at 2:00. Leaks were less commonly observed at lateral positions (3:00 and 9:00). The remaining 30 (73.2%) patients had leaks ≥3 mm, with clustering in the inferior-posterior region. The most common site was 7:00 9 (30.0%), followed by 6:00, 8:00, and 11:00 each 4 (13.3%).

In this multicentre core laboratory analysis, we report the first real-world standardized characterization of PDL spatial distribution using clock-face mapping on cardiac CT and TEE, with the posterior location being the major site of ≥3 mm PDLs across both modalities. Published data have focused on PDL size rather than location as a prognostic factor, demonstrating a graded association between increasing leak size on TEE and thromboembolic risk across different thresholds.^4^ Moreover, despite an apparent spatial pattern of clinically relevant large leaks, no prior studies have compared clinical outcomes by leak location, and the prognostic implications of regional PDL remain unknown.

Several factors can contribute to posterior vulnerability. Ostial ellipticity relative to the circular device design, which may create regional gaps, anatomical variability of the posterior ridge, and variable ostial angulation can impair device-tissue apposition and contribute to incomplete sealing.^5,6^ Current guidelines endorse TEE or CT at 45–90 days for imaging surveillance, with therapy individualized to bleeding risk and imaging findings.^7,8^

Our findings demonstrate a unified cross-modality pattern of posteriorly (TEE) and posterior-inferiorly (CT) clustered large leaks (≥3 mm), while smaller leaks (<3 mm) showing relative anterior tendency. These findings are consistent with a prior single-centre TEE study that localized PDLs (39/45) to the posterior 6:00–12:00 segment in 101 consecutive Watchman device implants.^3^ On the other hand, in an analysis of consecutive patients undergoing LAAO with Amplatzer devices, Korsholm et al. reported that 86% of PDLs (180/210) were located in the inferoposterior region.^6^ Together, these observations reinforce the concept that posterior vulnerability to these leaks is device independent and likely reflects underlying regional anatomical factors and device apposition mechanics rather than imaging artefacts.

The present large multicentre study provides a standardized and reproducible assessment of PDL distribution. The use of a unified clock-face mapping system across both TEE and CT enables a consistent framework for anatomical localization, enabling cross-modality comparison and improving interpretability. Additionally, inclusion of contemporary WATCHMAN FLX and FLX Pro devices enhances the relevance and generalizability of our findings to current clinical practice. Accordingly, a thorough spatial assessment of PDLs offers a framework that may carry substantial clinical implications by improving procedural planning, cross-modality comparisons, device refinement, medication adjustment, and closer patient surveillance. This may also help guide operators to pay a special attention to vulnerable regions during implantation.

This study is limited by its retrospective design. Moreover, the TEE and CT cohorts were not paired, precluding any patient-level concordance analysis between imaging modalities. Additionally, TEE assessment was predominantly performed using 2D imaging in a site-specific, non-standardized manner, without a mandated protocol for specific views or imaging planes, which may have affected PDL detection. The measurement methodologies differ between modalities: colour Doppler reflects jet width and is subject to gain and Nyquist limit variability, whereas CT measures the anatomical gap width directly; this may introduce systematic classification differences particularly near the 3 mm threshold. Patient-level longitudinal data were not available to assess temporal PDL evolution within our cohort. Clinical outcome data, including device-related thrombus and thromboembolic events, were not captured within this imaging registry. Finally, longer-term follow-up is needed to determine whether the observed spatial distribution of PDL translates into differences in thromboembolic risk or device-related thrombosis.

In conclusion, this multicentre core-laboratory analysis using a standardized clock-face mapping across both TEE and cardiac CT offers a reproducible and intuitive framework for spatial assessment of PDLs and may guide procedural planning and follow-up imaging strategies.

## Data Availability

The data used can be made available upon reasonable request.
